# Statins in the Treatment of Hepatitis C

**DOI:** 10.5812/hepatmon.5998

**Published:** 2012-06-30

**Authors:** Cristin Constantin Vere, Costin Teodor Streba, Liliana Streba, Ion Rogoveanu

**Affiliations:** 1 Internal Medicine and Gastroenterology Department, University of Medicine and Pharmacy of Craiova, Craiova, Romania

**Keywords:** Hepatitis C, Antiviral, Therapy

Hepatitis C virus (HCV) infection is an important health issue because it affects about 3% of the world population.It is estimated that 65–80% of HCV infections progress to chronic disease, and more than 20–50% of patients with chronic hepatitis develop hepatic cirrhosis. Hepatocellular carcinoma complicates 5% of the chronic hepatitis C cases [1][2]. Combination therapy with peginterferon-ribavirin, which is currently the standard treatment for chronic hepatitis C, shows a sustained virological response in 55% of patients. The remaining 45% of patients do not respond to antiviral therapy and are at risk of the HCV infection progressing to hepatic cirrhosis and hepatocellular carcinoma [3]. In addition, because of the numerous adverse effects and possible contraindications to the use of combination therapy with peginterferon and ribavirin, the administration of this antiviral therapy is limited to only some patients with chronic hepatitis C [4]. Because of these limitations, it is necessary to discover new therapeutic agents for eradicating HCV infection. Several recent studies have shown that statins, commonly used as cholesterol-lowering medication, can inhibit the replication of HCV [3][5][6]. Ikeda et al. [2] have reported in their 2006 study that statins can inhibit in vitro HCV replication. Using OR6 cells infected with HCV, the authors evaluated the antiviral activity of 5 statins: atorvastatin, fluvastatin, lovastatin, pravastatin, and simvastatin. All statins, except pravastatin, that were tested as monotherapy inhibited viral replication. Fluvastatin exhibited the strongest antiviral effect, whereas atorvastatin and simvastatin showed moderate inhibitory effects, and lovastatin exhibited a weak inhibitory effect on HCV replication. All of these statins exert a cholesterol-lowering effect by inhibiting the activity of 3-hydroxy3-methyl-glutaryl coenzyme A (HMG-CoA) reductase, an enzyme involved in cholesterol synthesis. Pravastatin, unlike the other tested statins, has no antiviral activity; hence, it inhibits HCV replication not by a direct action on HMG-CoA reductase, but by a specific antiviral mechanism [3]. In order to emphasize the presence of an existing antiviral mechanism, the authors showed that the antiviral activity of statins is not due to hepatotoxicity; they proved so by demonstrating that HCV replication is not inhibited by the destruction of hepatocytes [3]. Several in vivo studies have shown that the administration of statins in patients with chronic HCV infection is safe and has no severe adverse effects [7][8][9]. For its replication, HCV requires a number of proteins, which are involved in cholesterol synthesis. Statins are considered to exert their antiviral activity by blocking these proteins. Thus,by inhibiting HMG-CoA reductase activity, statins reduce the intracellular concentration of geranylgeranyl pyrophosphate, which lowers the level of mevalonate necessary in cholesterol synthesis. A strong argument in favor of this theory is the fact that adding geranylgeraniol and mevalonate in cell cultures treated with statins leads to the restoration of viral replication.

In the same study, Ikeda et al. [2] evaluated the possibility of replacing ribavirin with statins in the treatment of chronic hepatitis C. The efficacy of each tested statin was evaluated in combination therapy with interferon. All the statins, except pravastatin, showed higher inhibition of HCV replication when used in combination with interferon than when used as monotherapy. Interferon–fluvastatin combination therapy had the strongest antiviral effect, and the authors consider that this treatment is superior to standard therapy with interferon and ribavirin [3]. Another recently published study [10], which evaluated in vitro anti-HCV activity of 5 statins, confirmed the Japanese authors’ findings. According to these authors, mevastatin and simvastatin administered as mono-therapy have the strongest antiviral activity; lovastatin and fluvastatin have a moderate antiviral effect, while pravastatin has no antiviral activity. In addition, mevastatin and simvastatin enhance the antiviral activity of interferon α and of drugs that inhibit viral replication; mevastatin also prevents or reduces resistance to therapy with viral replication inhibitors [10]. Results of in vivo studies are controversial. According to the Initiating Dialysis Early and Late (IDEAL) study published in 2009, statins combined with peginterferon–ribavirin therapy significantly increase the sustained virological response rate, which is independent of serum lipid levels before the initiation of antiviral therapy. The findings of this retrospective study were not conclusive because of the small sample size; only 66 of the 3070 patients infected with HCV genotype 1 received treatment with statins [11]. Another prospective study [12] on 31 patients with chronic hepatitis C infection has shown that fluvastatin alone (in commonly used doses) has a moderate, variable, and short-term antiviral effect. However, the findings of the in vivo studies were not consistent with those of the in vitro studies. In 2007, o’Leary et al. published a prospective study on 10 patients and reported that atorvastatin (in commonly used doses) does not inhibit viral replication [13]. In 2009, Forde et al. [4] published a retrospective study including 6463 HCV seropositive patients; this study showed that statins do not inhibit viral replication in vivo. According to these authors, the discrepancy between the results of the in vivo and in vitro studies may be attributable to several factors; one such factor is the low level of statins in the serum than in the cell cultures, because of the pharmacokinetic properties of statins. All statins, except pravastatin, are largely retained in the first pass through the liver. The in vitro anti-HCV action may be because of an adaptation of the cell cultures that makes the cells sensitive to interferon action and increases their sensitivity to statins. As an adaptive mechanism, HCV could undergo in vivo mutations and become resistant to statins; paradoxically, statins could exert proviral effects by promoting the expression of low-density lipoprotein (LDL) receptors, thereby facilitating HCV penetration into noninfected hepatocytes [4]. Further, the same study showed that triglyceride-reducing agents, such as niacin, may have anti-HCV activity in vivo. There may be a direct relationship between triglyceride level and virus titer, considering that triglycerides metabolism is an intermediate step in the life cycle of HCV. The virus is released together with very low-density lipoprotein (VLDL) particles, and therefore, drugs such as niacin inhibit viral replication by lowering serum triglyceride levels [14][15][16].

Elucidating the role of statins in the treatment of chronic hepatitis C is important, and can be achieved by conducting prospective studies in large groups of patients. extensive research is required to determine the effectiveness of triglyceride-reducing agents for inhibiting HCV replication. Until the approval of new therapeutic agents, the peginterferon–ribavirin combination represented the therapy of choice for chronic viral hepatitis C. Recent data have shown that the addition of protease inhibitors such as boceprevir or telaprevir to the classic peginterferon-ribavirin combination therapy decreases the duration of treatment, thereby reducing the cost of treatment and the number of adverse effects [17][18]. Further, an increase of up to 30% was observed in the sustained virological response rates in non-responders, with naïve and genotype 1 HCV infection, and a corresponding increase of up to 40–50% was observed in patients showing relapse [19][20]. These recent advances have shown that statins play a role in improving treatment outcome and increasing the quality of life of HCV patients; however, the exact mechanism underlying their role is yet to be determined.
